# The Successor of Prof. James P. White

**Published:** 1882-01

**Authors:** 


### The Successor of Prof. James P. White.

We are happy to announce that Dr. M. B. Mann has been selected to fill the vacancy in the staff of the Medical College, caused by the death of Prof. White. He delivered his first lecture on the 27th ult. His style is quiet and impressive, his reasoning close and argumentative, his voice is clear and distinct. He was evidently quite at home with his subject, and by a persuasive, conversational, didactic manner, without any attempt at oratory, conveyed his instruction in the way best calculated to make it understood and remembered. Altogether he made a very favorable impression. Dr. Mann is about thirty-six years of age, he was for three years the assistant of the eminent Dr. Thomas, of New York, who recommended him very highly for the position, to which he has been called. Dr. Mann has been the Professor of Gynecology in the medical school of Yale college for several years, and has practiced exclusively as a specialist in diseases of women, at Hartford, which city has been his residence during his connection with Yale. He has left a successful and lucrative business, to come to a larger field both of teaching and population. He does not design to engage in general practice, but will confine himself to consultations in midwifery, and to the treatment of gynecological disorders. He will bring his family here in the spring. In the meantime he has taken offices at No. 7 W. Chippewa street. We are glad of his accession to the medical profession of this city and extend to him a most cordial welcome.

